# Preventing Cryptococcosis—Shifting the Paradigm in the Era of Highly Active Antiretroviral Therapy

**DOI:** 10.1007/s40475-015-0045-z

**Published:** 2015-04-21

**Authors:** David Meya, Radha Rajasingham, Elizabeth Nalintya, Mark Tenforde, Joseph N Jarvis

**Affiliations:** Infectious Disease Institute, College of Health Sciences, Makerere University, Mulago Hill Road, # 22418, Kampala, Uganda; Department of Medicine, Center for Infectious Diseases and Microbiology Translational Research, University of Minnesota, 450 Delaware Street, MMC250, Minneapolis, MN 55455 USA; School of Medicine, College of Health Sciences, Makerere University, Kampala, Uganda; Botswana-UPenn Partnership, P.O. Box AC 157 ACH, Gaborone, Botswana; Division of Infectious Diseases, Department of Medicine, Perelman School of Medicine, University of Pennsylvania, Philadelphia, PA USA; London School of Hygiene and Tropical Medicine, London, UK

**Keywords:** HIV, Cryptococcosis, Cryptococcal antigen, Fluconazole, CD4, CRAG screening, Preemptive therapy

## Abstract

Cryptococcosis remains a significant cause of morbidity and mortality among HIV-infected patients, especially in sub-Saharan Africa where it causes up to 20 % of AIDS-related deaths in HIV programs. A new, highly sensitive, and affordable point of care diagnostic test for cryptococcal infection, the lateral flow assay, can detect early sub-clinical cryptococcosis especially in areas with limited laboratory infrastructure. With a prevalence of detectable sub-clinical cryptococcal infection averaging 7.2 % (95 % CI 6.8–7.6 %) among 36 cohorts with CD4 <100 cells/μL in Africa, together with data showing that preemptive fluconazole prevents overt cryptococcal disease in this population, implementing a screen and treat strategy as part of HIV care practice among patients with CD4 <100 cells/μL could prevent the incidence of often fatal cryptococcal meningitis in the setting of the HIV pandemic.

## Introduction

One of the most significant contributors to the increased burden of cryptococcosis worldwide is indisputably HIV infection. With 35.3 million individuals living with HIV and an estimated peak of 2.3 million HIV-associated deaths in 2012 [1], the rise in the incidence of opportunistic infections has been remarkable, resulting in tremendous strain on healthcare resources and lost lives and income especially in low- and middle-income countries (LMICs).

Prior to the global HIV epidemic, cryptococcosis was a disease primarily of individuals with low immunity, particularly after long-term use of immunosuppressive drugs and solid-organ transplantation, with other non-HIV risk factors including hematopoietic or other malignancies, innate immune defects, advanced liver or renal disease, sarcoidosis, rheumatologic disease, and diabetes mellitus [2–9]. One of the initial indicators of the HIV epidemic was the increase in cases of cryptococcal meningitis (CM) [10–12]. Although the incidence of CM has decreased in high-income countries with the advent of antiretroviral therapy (ART), sub-Saharan Africa continues to grapple with a high prevalence of HIV and opportunistic infections, with cryptococcal meningitis as the leading cause of meningitis among HIV-infected adults [3, 13, 14]. Over the last 3 decades of the HIV epidemic, efforts have been invested in improving cryptococcal care with revision of national guidelines focusing on diagnosis and management of cryptococcal meningitis but less so on prevention.

Fluconazole prophylaxis given to HIV-infected patients with CD4 <200 cells/μL before or during ART has been shown to reduce the risk of developing cryptococcal meningitis, including using thrice-weekly fluconazole dosing [15•, 16]. However, this strategy is not as cost-effective as targeted cryptococcal antigen (CRAG) screening followed by preemptive therapy using fluconazole in the setting of cryptococcal antigenemia [17•, 18•]. Although currently only recommended in HIV-infected persons, it is also possible that CRAG screening and preemptive fluconazole therapy would be beneficial in solid-organ transplantation patients.

Preventing new HIV infections, early HIV diagnosis, implementing early linkage to care, ensuring timely initiation of ART with strict adherence, and adequate patient follow-up along the continuum of HIV care would ideally prevent incident cryptococcosis and other opportunistic infections. In this review, we outline the strategies for preventing cryptococcosis in the context of HIV infection and discuss the impact of new cryptococcal diagnostics on these strategies.

## Burden of Cryptococcal Disease

Cryptococcus is an encapsulated yeast acquired by inhalation that can disseminate to cause a severe meningoencephalitis primarily in people with defective cell-mediated immunity. *Cryptococcus neoformans* is responsible for most human disease and has a worldwide distribution with an ecological niche in decaying organic matter and soil containing bird excrement [19].

In high-income country (HIC) settings, including the USA, Western Europe, and Australia, cryptococcosis emerged during the HIV epidemic in the 1980s as a cause of meningitis in 5–10 % of HIV-infected individuals [20, 21]. Rollout of ART starting in 1997 led to a dramatic decline in cases, illustrated by a greater than 90 % decreased incidence in a large UK cohort in 2006–2007 compared to 1996–1997 [14]. A review of over 200 million US hospital admissions from 1997 to 2009 similarly found a 5.8 % annual decline of HIV-associated CM and proportional rise in non-HIV CM over this period from 16 to 29 % of all cases (major risk factors including solid-organ transplant and long-term use of corticosteroids or other immunosuppressant drugs) [3]. Early case-fatality rate of CM in HICs with access to amphotericin-based induction therapy (±flucytosine) is as low as 10 % in clinical trials, when excluding lost to follow-up [22, 23•]. This decline is yet to be demonstrated in LMICs.

The vast majority of cryptococcal meningitis cases and deaths occur in LMICs with high HIV prevalence coupled with common delays in HIV diagnosis and ART initiation. Recent data suggests that well over 90 % of cryptococcal meningitis cases in LMICs are in people infected with HIV [13, 24–26]. In 2006, of an estimated 957,900 HIV-associated CM cases and 624,725 deaths worldwide, more than 90 % of cases and 95 % of deaths occurred in sub-Saharan Africa (720,000 cases, 504,000 deaths), South and Southeast Asia (120,000 cases, 66,000 deaths), and Latin America (54,400 cases, 29,900 deaths) [23•]; however, these estimates are currently under revision. Cryptococcus is the leading cause of meningitis in much of Southern and Eastern Africa and causes up to 20 % of deaths in HIV-infected cohorts from sub-Saharan Africa [13, 27–29]. Health facilities in LMICs often lack amphotericin-based induction therapy (instead using high-dose fluconazole) and are under-resourced for performing serial lumbar punctures and monitoring common side effects of antifungal treatment, all of which contribute to poor treatment outcomes. Park et al. previously estimated a 3-month case-fatality rate in LMICs at 55 and 70 % in sub-Saharan Africa [23•]. More recent reports suggest continued poor treatment outcomes, with *in*-*hospital* mortality of 27.9 % in a study from Burkina Faso, 52.0 % in Cameroon, 48.1 % in Ethiopia, 50.0 % in India, 35.8 % in Kenya, 62.2 % in Senegal, and 40.5 % in South Africa [24, 26, 30–34].

## Asymptomatic Cryptococcosis

Cryptococcosis is one of the few infectious diseases in which the presence of disseminated infection can be demonstrated even while patients remain asymptomatic. Asymptomatic cryptococcosis (positive serum CRAG with absent or minimal symptoms) is a sub-clinical infectious state known to precede clinically apparent disease by weeks to months and is strongly associated with risk of incident meningitis and all-cause mortality [35, 36•, 37]. Cryptococcal antigenemia is common in persons with AIDS and is inversely related to CD4 count (Table 1) [41, 43, 44, 49, 54]. In sub-Saharan Africa, patients with CD4 ≤100 cells/μL have a CRAG prevalence reported between 2.2 and 21.0 % or up to 11.5 % in studies including only asymptomatic, ART-naïve patients [35, 36•, 38, 39•, 40–47, 55].

**Table 1:** Prevalence of Cryptococcal antigenemia in HIV-infected patients with CD4 ≤100 cells/μL

Region	Country	Setting	Year	Prevalence	No Hx Crypto	ASx	Other information	Test	Reference
Africa									
Cape Town	South Africa	Outpatient	2002–2005	6.7 % (21/312)	Yes	Yes	ART naive	LA	[36•]
Mbarara	Uganda	Inpatient and outpatient	∼2003	10.7 % (21/197)^a^	NS	No	ART naïve; 61.9 % (13/21) CRAG+ patients had confirmed CM	LA	[38]
Tororo	Uganda	Outpatient	2003–2004	5.8 % (22/377)	Yes	Yes	ART naive	LA	[35]
Kampala	Uganda	Outpatient	2004–2006	8.8 % (26/295)	Yes	Yes	ART naive	LA	[39•]
Kusami	Ghana	Outpatient	2008–2009	2.2 % (2/92)	No	Yes	% on ART NS but likely low (78 % samples tested within 1 week of HIV diagnosis)	LA	[40]
Kampala	Uganda	Inpatient and outpatient	2009–2010	18.8 % (69/367)	Yes	No	ART naïve; of 30 patients who consented to LP, 24 had confirmed CM (≥34.8 % of all CRAG+ patients)	LA	[41]
Rongo and Kisumu	Kenya	Outpatient	2010–2011	11.5 % (59/514)	No	Yes	ART naïve	LA	[42]
Addis Ababa	Ethiopia	Outpatient	2011	11.2 % (13/116)	Yes	No	68 % on ART	LA	[43]
Benin City	Nigeria	Outpatient	2011	21.0 % (17/81)	Yes	NS	ART naïve	LA	[44]
Moshi	Tanzania	Outpatient	2011–2012	4.8 % (6/124)	Yes	Yes	ART naïve (or on ART <6 months)	LA / LFA	[45]
Mwanza	Tanzania	Outpatient	2012–2013	8.2 % (6/73)	Yes	Yes	ART naive	LFA	[46]
Ekurhuleni and Johannesburg	South Africa	Outpatient	2012–2014	4.5 % (839/18,544)	No	No	% on ART unknown	LFA	[47]
Asia									
Bangkok	Thailand	Outpatient	2003–2007	12.9 % (11/85)	Yes	Yes	ART naive	LA	[48]
Phnom Penh	Cambodia	Inpatient and outpatient	2004	20.6 % (58/282)	Yes	No	ART naive	LA	[49]
Multi-site	Thailand	Outpatient	2005–2007	10.7 % (9/84)	Yes	NS	ART naive	LA	[50]
Bandung	Indonesia	Outpatient	2007–2011	7.1 % (58/810)	Yes	Yes	ART naive	LFA	[51]
Hanoi and Ho Chi Minh City	Vietnam	Outpatient	2009–2012	4.0 % (9/226)	Yes (in part of cohort)	NS	ART naïve	LFA	[52]
Europe									
London	UK	Inpatient	2004–2010	5.0 % (8/157)	No	No	ART naïve; 7/8 CRAG-positive patients with CM	LA	[53]
USA									
Multi-site	USA	Outpatient	1986–2012	2.9 % (55/2872)	NS	NS	% on ART NS	LFA	[54]

Adapted from Meyer AC and Jacobson MA 2013 23715897

*ART* antiretroviral therapy, *Asx* asymptomatic, *CM* cryptococcal meningitis, *CRAG* cryptococcal antigen, *HIV* human immunodeficiency virus, *Hx* history, *LA* latex agglutination, *LFA* lateral flow assay, *LP* lumbar puncture, *NS* not specified

^a^CRAG prevalence limited to patients with CD4 count <50 cells/μL

In Southeast Asia, CRAG prevalence in patients with CD4 ≤100 cells/μL is reported between 4.0 and 20.6 % or up to 12.9 % in studies including only asymptomatic, ART-naïve patients [48–52]. In untreated asymptomatic antigenemic patients starting ART, over 25 % go on to develop incident cryptococcal meningitis within 12 months versus as few as 0 % of CRAG-negative patients [36•]. Baseline cryptococcal antigenemia is also a strong independent predictor of mortality in the first year of ART after adjusting for baseline clinical (e.g., CD4 count) and demographic factors, with a study from Indonesia, including only patients without initial symptoms of meningitis, showing an adjusted hazard ratio for death of 2.6 (95 % CI 1.4–4.6) and a study from South Africa showing an adjusted hazard ratio of 3.2 (95 % CI 1.5–6.6) [36•, 51]. A Ugandan study of ART-naïve, TB/HIV co-infected patients followed for 2 years found an adjusted hazard ratio for death of 4.27 (95 % CI 1.5–12.1) in those who were CRAG positive at baseline [56]. For pre-ART asymptomatic CRAG-positive patients, another Ugandan study found a relative risk of death (within 12 weeks after ART initiation) of 6.6 (95 % CI 1.9–23.6) compared to pre-ART CRAG-negative patients [35].

Robust evidence is lacking to guide the optimal management of patients found to have asymptomatic cryptococcal antigenemia, but expert guidelines suggest using 800 mg of fluconazole for 2 weeks followed by 400 mg for 8 weeks [57]. These guidelines have been adopted with some variation by several countries (Table 2).

**Table 2:** Countries that have revised their national guidelines following the WHO 2011 rapid advice and the individual fluconazole regimens proposed

Country	Screening recommendations	Fluconazole regimen	Comments on implementation
Rwanda [58] (2011 guidelines)	Patients with advanced immunosuppression are at higher risk than others of having an asymptomatic or symptomatic cryptococcal infection; therefore, it is recommended to screen for cryptococcal disease in every patient with CD4 <200 cells/μL using CRAG testing on plasma	Fluconazole 800–1200 mg daily for 2 weeks as an induction phase for patients with asymptomatic cryptococcal infection (with antigenemia only)	None
Kenya [59] (2011 guidelines)	CRAG screening is recommended in PLHIV prior to starting ART if CD4 is <100 cells/μL	No mention is made of the preemptive therapy to be used after screening	It is not possible (and neither desirable) for *all* facilities providing ART services to perform all the laboratory tests required for HIV care and treatment. If a facility does not have on-site capacity to carry out any particular test, arrangements should be made to transport specimens to a local or regional reference laboratory. Facilities are encouraged to join or form regional networks of laboratory services to improve access to these tests
Botswana [60] (2012)			No mention is made of CRAG screening
Zimbabwe [61] (2013)	Screening of asymptomatic ART-naïve individuals with CD4 count <100 cells/μL is recommended and should be done with a CRAG test using latex agglutination tests (LA) or lateral flow assays (LFA) on serum, plasma, or CSF. A lumbar puncture should be offered to individuals who screen positive for cryptococcal antigen, as a positive cryptococcal antigen may precede the onset of clinical cryptococcal meningitis by many weeks	Fluconazole 800 mg daily for 2 weeks, then fluconazole 400 mg daily for 8 weeks, followed by maintenance therapy with fluconazole 200 mg daily until CD4 >200 cells/μL for 6 months	Serum CRAG positive: if available recommend LP; If CSF CRAG positive, manage for cryptococcal meningitis; if CSF CRAG negative, treat with fluconazole Timing of ART for individuals with asymptomatic cryptococcal antigenemia is unknown. We recommend initiation of ART 2–4 weeks after initiation of antifungal therapy in individuals who screen positive for serum CRAG without any evidence of disseminated cryptococcal meningitis
Uganda [62] (2013 guidelines)	All PLHIV with CD4 <100 cells/μL should be screened for cryptococcal antigen using serum or plasma irrespective of symptoms	Fluconazole 800 mg daily for 2 weeks followed by 400 mg daily for 8 weeks	CRAG screening is to be done either by CRAG LA or LFA (on serum or plasma) Initiate ART 2 weeks after staring preemptive therapy
South Africa [63] (2015 guidelines)	HIV-infected adults with CD4^+^ T lymphocyte count <100 cells/μL	Fluconazole 800 mg daily for 2 weeks as outpatient, fluconazole 400 mg daily for 2 months then 200 mg daily. Continue fluconazole for minimum of 1 year in total and discontinue when patient has had 2 CD4 counts >200 cells/μL taken at least 6 months apart	Screen for cryptococcal antigenemia on serum or plasma by reflex laboratory or clinician-initiated testing. If clinician-initiated testing is performed, screening should be restricted to ART-naive adults with no prior CM. Either the LA or LFA may be used as a screening test
USA [64]			Although fluconazole and itraconazole have been shown to reduce frequency of primary cryptococcal disease among those who have CD4 cell counts <100 cells/μL, primary prophylaxis for cryptococcosis is not routinely recommended in this group with advanced medical care. This recommendation is based on the relative infrequency of cryptococcal disease, lack of survival benefits, possibility of drug-drug interactions, creation of direct antifungal drug resistance, medication compliance, and costs

Ethiopia and Mozambique have also incorporated CRAG screening into revised HIV care guidelines

## The Changing Landscape of Cryptococcal Diagnostics

Historically, cryptococcal infection has been diagnosed by India ink microscopy, latex agglutination for cryptococcal antigen, or culture. Culture is ultimately considered to be the gold standard; however, delays in obtaining a result make culture clinically unhelpful for initial management decisions. CRAG assays include enzyme immunoassay (EIA), latex agglutination (LA), or a recently developed point-of-care lateral flow assay (LFA) (IMMY, Inc., Norman, Oklahoma), which was approved by the US Food and Drug Administration for use with CSF or serum in July 2011. The test utilizes an immunochromatographic test strip containing gold-conjugated monoclonal antibodies that bind to glucuronoxylomannan (GXM) cryptococcal antigen and is capable of detecting all cryptococcal serotypes (A-D). One drop (∼40 μL) of the patient sample is mixed with one drop of diluent and a binary readout (two bands—test and control—for a positive test) is produced within 10 min if antigen is detected. The assay has a number of qualities that make it ideal for diagnostic testing in resource-limited settings, including low cost, high sensitivity/specificity, point-of-care testing, stability of diluent and test strips at room temperature with a long shelf life (up to 2 years), minimal training requirement, and no need for processing of samples (e.g., pretreatment, heat inactivation) or specialized laboratory equipment. Furthermore, titers can be done using this assay for CRAG quantification.

Using serum or plasma samples, the LFA has excellent sensitivity and specificity and a high agreement with results from other antigen-based tests [45, 65, 66•, 67, 68]. Serum LFA had a sensitivity of 99.6 to 100 % in published studies including patients with laboratory-confirmed cryptococcal disease and a specificity of 92 % in a study of patients hospitalized with suspected meningitis (although this may be an understatement as several of the “false positives” on LFA may have actually been false negatives on reference tests) [65, 69, 70•, 71]. Plasma LFA testing appears to perform equivalently to serum testing [70•, 71]. Additionally, serum LFA and LA had a 100 % agreement in a study from Tanzania evaluating CRAG prevalence in asymptomatic, ART-naïve patients, supporting LFA as a good alternative to other antigen tests for use in CRAG screening for prevention [45]. Unpublished data from Uganda and South Africa suggests that there is 100 % agreement between whole blood, serum, and plasma CRAG LFA testing, demonstrating that fingerstick is a viable option for bedside detection of CRAG, particularly in settings where phlebotomy is unavailable [72].

The LFA performs sub-optimally, however, with urine and saliva samples. Although sensitive, LFA in urine lacks specificity, leading to a poor positive predictive value for cryptococcosis [46, 65, 69, 70•, 71]. Attempts to improve the specificity of the urine assay by altering the diluent have been unsuccessful, leading to a reduction in test sensitivity compared to serum LFA as reference and continued sub-optimal specificity (sensitivity decreasing from 100 % to 80 % and specificity increasing from 73.8 % to 91.5 %) [46]. Using samples of saliva, a recent Ugandan study found excellent specificity but poor sensitivity (88 % in symptomatic patients and only 27 % in asymptomatic patients) of the LFA in detecting cryptococcal antigenemia using serum/plasma samples for reference [73]. In a review of the published literature addressing performance of the LFA assay, median CSF sensitivity was 100 %, and median specificity was 97.7 %. In serum, median sensitivity was again 100 %, with median specificity of 99.5 % [74].

## Cost-Effectiveness of CRAG Screening

The cost per sample using the CRAG LFA is substantially lower than other antigen test methodologies or culture. The manufacturer offers the test for US$2 per sample in resource-limited settings with a current delivered in-country real world cost from local distributors of US$3–4. Further efficiencies in distribution are possible. The cost in high-income countries is $5 per LFA. Cost-effectiveness analyses of a CRAG “screen and treat” approach in antiretroviral therapy initiators with CD4 counts <100 cells/μL have been performed in varied settings, including Cambodia, South Africa, Uganda, and Vietnam [17•, 18•, 39•, 52]. All studies have shown this approach to be extremely cost-effective, even using a low-range prevalence estimate of 2 %. As these studies have CRAG testing cost between US$4.13 per test and US$16.75 per test, as a screen and treat strategy, the LFA at US$2.50 per test would further increase cost-effectiveness.

## Costs of Cryptococcal Meningitis Management

In comparison to the costs of screening, the costs of management of symptomatic cryptococcal meningitis are substantially greater. Costs of cryptococcal meningitis vary by country but include costs of medications, healthcare personnel for 2-week inpatient hospitalization, laboratory monitoring for medication toxicity, and finally costs of serial lumbar punctures including manometers if available. In Uganda, total cost of medical care for 2 weeks of induction therapy with amphotericin and flucytosine is estimated at $467.48 [75•]. Given that flucytosine is unavailable, the total cost of care with what is considered the next best regimen of amphotericin + fluconazole is $402.07, with the cost of medication making up a small percentage of this total cost (23 %). In South Africa, the estimated cost for care of cryptococcal meningitis is $2883 [18•]. This variability is likely due to much higher cost of personnel in South Africa. Estimated US costs for 14 days of inpatient management of cryptococcal meningitis is $50,000 with flucytosine alone costing approximately $9000 for a 70-kg adult [76].

## Integrating Cryptococcal Antigen Screening and Fluconazole Therapy into Routine Care—Challenges and Opportunities

Integrating CRAG screening into routine HIV care could potentially provide a platform for improved linkage to care, promoting continuity in care while diminishing attrition, and contributing to improved patient outcomes. As an integrated practice, healthcare facilities could focus on the more severely immunosuppressed patients, by having dedicated staff to follow up CD4 and CRAG test results, and ensure that this population of patients (with CD4 <100 cells/μL) returns to the clinic in a timely manner to receive the appropriate intervention, including fluconazole, if CRAG positive. The timing of ART initiation among patients with asymptomatic cryptococcal antigenemia has not been studied; however, current guidelines recommend initiating ART after the 2 weeks of high-dose fluconazole. Following the release of the 2011 WHO rapid advice guidelines on the diagnosis, prevention, and management of cryptococcal disease in HIV-infected individuals, several countries have revised their national guidelines to reflect these changes; however, actual implementation of these guidelines has been varied (Table 2) [57].

Several operational challenges must be addressed as screen and treat programs are implemented. First, cryptococcal screening must be integrated into routine management algorithms for patients who require rapid ART initiation. Second, for the program to succeed, patients found to have cryptococcal antigenemia must be immediately traced, assessed, and initiated on antifungal treatment before they develop meningitis or die. Laboratory reporting and clinic tracing systems need to be enhanced. Third, supply, procurement, and distribution of CRAG tests and antifungal drugs to clinics must be adequate and consistent, and nurses who identify patients with antigenemia need to have the ability to initiate antifungal treatment.

From the experience of an ongoing CRAG screening study in Kampala Uganda, challenges of rolling out the CRAG screening program include lack of adequate staff training across all levels of health cadres, from laboratory, clinical, and administrative staff, which limits the appreciation of program ownership; lack of adequate logistics and the irregular supply of CD4 testing reagents, which slows the screening process; lack of CRAG screening tests at health facilities; lack of a steady supply of fluconazole for preemptive treatment; and, finally, poor referral systems in the absence of relevant manpower to handle this very sick population. More often than not, health facilities are ill equipped with the required drugs and tests to manage patients with breakthrough meningitis.

## Conclusions

Significant improvement in cryptococcal diagnosis has occurred along with a better understanding of cryptococcal management. By integrating CRAG screening and fluconazole preemptive therapy as recommended by the WHO, the morbidity and mortality occasioned by cryptococcal disease could be drastically reduced. In the long term, with a subsiding number of newly diagnosed HIV-infected individuals presenting to HIV care with lower CD4 counts, increasing numbers of patients started on ART at CD4 counts >500 cells/μL, and improved linkage to long-term HIV care, screen and treat programs will become less expensive with fewer persons requiring CRAG testing and fluconazole therapy. There still remains the benefit of saving money to preemptively treat patients who would otherwise develop cryptococcal meningitis. Ideally, with perfect CRAG screening programs, one could envision elimination of cryptococcal meningitis using this screen and treat strategy, especially in sub-Saharan Africa. However, challenges remain on how best to implement such a strategy and a better understanding of risk factors for breakthrough cryptococcal meningitis remains to be elucidated.

## References

[1] Maartens G, Celum C, Lewin SR (2014). HIV infection: epidemiology, pathogenesis, treatment, and prevention. Lancet.

[2] Zhu LP, Wu JQ, Xu B, Ou XT, Zhang QQ, Weng XH (2010). Cryptococcal meningitis in non-HIV-infected patients in a Chinese tertiary care hospital, 1997–2007. Med Mycol.

[3] Pyrgos V, Seitz AE, Steiner CA, Prevots DR, Williamson PR (2013). Epidemiology of cryptococcal meningitis in the US: 1997–2009. PLoS One.

[4] Pappas PG, Perfect JR, Cloud GA, Larsen RA, Pankey GA, Lancaster DJ (2001). Cryptococcosis in human immunodeficiency virus-negative patients in the era of effective azole therapy. Clin Infect Dis.

[5] Pappas PG, Alexander BD, Andes DR, Hadley S, Kauffman CA, Freifeld A (2010). Invasive fungal infections among organ transplant recipients: results of the Transplant-Associated Infection Surveillance Network (TRANSNET). Clin Infect Dis.

[6] Neofytos D, Fishman JA, Horn D, Anaissie E, Chang CH, Olyaei A (2010). Epidemiology and outcome of invasive fungal infections in solid organ transplant recipients. Transpl Infect Dis.

[7] Kiertiburanakul S, Wirojtananugoon S, Pracharktam R, Sungkanuparph S (2006). Cryptococcosis in human immunodeficiency virus-negative patients. Int J Infect Dis.

[8] Brizendine KD, Baddley JW, Pappas PG (2013). Predictors of mortality and differences in clinical features among patients with Cryptococcosis according to immune status. PLoS One.

[9] Bernard C, Maucort-Boulch D, Varron L, Charlier C, Sitbon K, Freymond N (2013). Cryptococcosis in sarcoidosis: cryptOsarc, a comparative study of 18 cases. QJM.

[10] Vandepitte J, Verwilghen R, Zachee P (1983). AIDS and cryptococcosis (Zaire, 1977). Lancet.

[11] Serwadda D, Mugerwa RD, Sewankambo NK, Lwegaba A, Carswell JW, Kirya GB (1985). Slim disease: a new disease in Uganda and its association with HTLV-III infection. Lancet.

[12] Levy RM, Bredesen DE, Rosenblum ML (1985). Neurological manifestations of the acquired immunodeficiency syndrome (AIDS): experience at UCSF and review of the literature. J Neurosurg.

[13] Jarvis JN, Meintjes G, Williams A, Brown Y, Crede T, Harrison TS (2010). Adult meningitis in a setting of high HIV and TB prevalence: findings from 4961 suspected cases. BMC Infect Dis.

[14] Garvey L, Winston A, Walsh J, Post F, Porter K, U. K. Collaborative HIV Cohort Study Steering Committee (2011). HIV-associated central nervous system diseases in the recent combination antiretroviral therapy era. Eur J Neurol.

[15.•] Parkes-Ratanshi R, Wakeham K, Levin J, Namusoke D, Whitworth J, Coutinho A, et al. Primary prophylaxis of cryptococcal disease with fluconazole in HIV-positive Ugandan adults: a double-blind, randomised, placebo-controlled trial. Lancet Infect Dis. 2011;11(12):933–41. doi:10.1016/S1473-3099(11)70245-6. **This article highlights the efficacy of fluconazole in preventing the incidence of cryptococcal meningitis in HIV-infected individuals**. 10.1016/S1473-3099(11)70245-6 PMC4856956 21982529

[16] Singh N, Barnish MJ, Berman S, Bender B, Wagener MM, Rinaldi MG (1996). Low-dose fluconazole as primary prophylaxis for cryptococcal infection in AIDS patients with CD4 cell counts of < or = 100/mm^3^: demonstration of efficacy in a positive, multicenter trial. Clin Infect Dis.

[17.•] Micol R, Tajahmady A, Lortholary O, Balkan S, Quillet C, Dousset JP, et al. Cost-effectiveness of primary prophylaxis of AIDS associated cryptococcosis in Cambodia. PLoS One. 2010;5(11):e13856. doi:10.1371/journal.pone.0013856. **This article shows that, although cryptococcal antigen screening followed by fluconazole treatment is more cost-effective than primary fluconazole prophylaxis, it is also less effective when the CD4 count is below 50 cells/μL**. 10.1371/journal.pone.0013856 PMC2976692 21085478

[18.•] Jarvis JN, Harrison TS, Lawn SD, Meintjes G, Wood R, Cleary S. Cost effectiveness of cryptococcal antigen screening as a strategy to prevent HIV-associated cryptococcal meningitis in South Africa. PLoS One. 2013;8(7):e69288. doi:10.1371/journal.pone.0069288. **This article compares the cost-effectiveness of 4 different strategies to prevent cryptococcal-related mortality among patients with CD4 <100 cells/μL, showing that the least costly strategy was CRAG screening followed by fluconazole when CRAG prevalence was ≥0.6 %**. 10.1371/journal.pone.0069288 PMC3716603 23894442

[19] Jarvis JN, Harrison TS (2007). HIV-associated cryptococcal meningitis. AIDS.

[20] Powderly WG (1993). Cryptococcal meningitis and AIDS. Clin Infect Dis.

[21] Bicanic T, Harrison TS (2004). Cryptococcal meningitis. Br Med Bull.

[22] van der Horst CM, Saag MS, Cloud GA, Hamill RJ, Graybill JR, Sobel JD (1997). Treatment of cryptococcal meningitis associated with the acquired immunodeficiency syndrome. National Institute of Allergy and Infectious Diseases Mycoses Study Group and AIDS Clinical Trials Group. N Engl J Med.

[23.•] Park BJ, Wannemuehler KA, Marston BJ, Govender N, Pappas PG, Chiller TM. Estimation of the current global burden of cryptococcal meningitis among persons living with HIV/AIDS. AIDS. 2009;23(4):525–30. doi:10.1097/QAD.0b013e328322ffac. **This article provides the only estimates of the burden attributed to cryptococcal disease worldwide and provides evidence for a need to intervene and reduce the high incidence of cryptococcal disease, especially in sub-Saharan Africa**. 10.1097/QAD.0b013e328322ffac 19182676

[24] Sow D, Tine RC, Sylla K, Djiba M, Ndour CT, Dieng T (2013). Cryptococcal meningitis in Senegal: epidemiology, laboratory findings, therapeutic and outcome of cases diagnosed from 2004 to 2011. Mycopathologia.

[25] Leal AL, Faganello J, Fuentefria AM, Boldo JT, Bassanesi MC, Vainstein MH (2008). Epidemiological profile of cryptococcal meningitis patients in Rio Grande do Sul. Brazil Mycopathologia.

[26] Bamba S, Lortholary O, Sawadogo A, Millogo A, Guiguemde RT, Bretagne S (2012). Decreasing incidence of cryptococcal meningitis in West Africa in the era of highly active antiretroviral therapy. AIDS.

[27] Lawn SD, Harries AD, Anglaret X, Myer L, Wood R (2008). Early mortality among adults accessing antiretroviral treatment programmes in sub-Saharan Africa. AIDS.

[28] Jarvis JN, Boulle A, Loyse A, Bicanic T, Rebe K, Williams A (2009). High ongoing burden of cryptococcal disease in Africa despite antiretroviral roll out. AIDS.

[29] Castelnuovo B, Manabe YC, Kiragga A, Kamya M, Easterbrook P, Kambugu A (2009). Cause-specific mortality and the contribution of immune reconstitution inflammatory syndrome in the first 3 years after antiretroviral therapy initiation in an urban African cohort. Clin Infect Dis.

[30] Mdodo R, Brown K, Omonge E, Jaoko W, Baddley J, Pappas P (2010). The prevalence, clinical features, risk factors and outcome associated with cryptococcal meningitis in HIV positive patients in Kenya. East Afr Med J.

[31] Luma HN, Tchaleu BC, Temfack E, Doualla MS, Ndenga DP, Mapoure YN (2013). HIV-associated central nervous system disease in patients admitted at the Douala General Hospital between 2004 and 2009: a retrospective study. AIDS Res Treat.

[32] Lessells RJ, Mutevedzi PC, Heller T, Newell ML (2011). Poor long-term outcomes for cryptococcal meningitis in rural South Africa. S Afr Med J.

[33] Berhe T, Melkamu Y, Amare A (2012). The pattern and predictors of mortality of HIV/AIDS patients with neurologic manifestation in Ethiopia: a retrospective study. AIDS Res Ther.

[34] Agarwal R, Kalita J, Marak RS, Misra UK (2012). Spectrum of fungal infection in a neurology tertiary care center in India. Neurol Sci.

[35] Liechty CA, Solberg P, Were W, Ekwaru JP, Ransom RL, Weidle PJ (2007). Asymptomatic serum cryptococcal antigenemia and early mortality during antiretroviral therapy in rural Uganda. Trop Med Int Health.

[36.•] Jarvis JN, Lawn SD, Vogt M, Bangani N, Wood R, Harrison TS. Screening for cryptococcal antigenemia in patients accessing an antiretroviral treatment program in South Africa. Clin Infect Dis. 2009;48(7):856–62. doi:10.1086/597262. **This article provides retrospective evidence that cryptococcal antigenemia is 100 % sensitive for predicting development of cryptococcal meningitis and is an independent predictor of mortality during the first year of ART**. 10.1086/597262 PMC2875173 19222372

[37] French N, Gray K, Watera C, Nakiyingi J, Lugada E, Moore M (2002). Cryptococcal infection in a cohort of HIV-1-infected Ugandan adults. AIDS.

[38] Tassie JM, Pepper L, Fogg C, Biraro S, Mayanja B, Andia I (2003). Systematic screening of cryptococcal antigenemia in HIV-positive adults in Uganda. J Acquir Immune Defic Syndr.

[39.•] Meya DB, Manabe YC, Castelnuovo B, Cook BA, Elbireer AM, Kambugu A, et al. Cost-effectiveness of serum cryptococcal antigen screening to prevent deaths among HIV-infected persons with a CD4+ cell count < or = 100 cells/microL who start HIV therapy in resource-limited settings. Clin Infect Dis. 2010;51(4):448–55. doi:10.1086/655143. **This article shows the survival benefit of using fluconazole therapy for HIV-infected patients with CD4 <100 cells/μL in a retrospective cohort with cryptococcal antigenemia as the basis for understanding the use of preemptive fluconazole to improve survival among patients with asymptomatic cryptococcal antigenemia**. 10.1086/655143 PMC2946373 20597693

[40] Mamoojee Y, Shakoor S, Gorton RL, Sarfo S, Appiah LT, Norman B (2011). Short communication: low seroprevalence of cryptococcal antigenaemia in patients with advanced HIV infection enrolling in an antiretroviral programme in Ghana. Trop Med Int Health.

[41] Oyella J, Meya D, Bajunirwe F, Kamya MR (2012). Prevalence and factors associated with cryptococcal antigenemia among severely immunosuppressed HIV-infected adults in Uganda: a cross-sectional study. J Int AIDS Soc.

[42] Meyer AC, Kendi CK, Penner JA, Odhiambo N, Otieno B, Omondi E (2013). The impact of routine cryptococcal antigen screening on survival among HIV-infected individuals with advanced immunosuppression in Kenya. Trop Med Int Health.

[43] Alemu AS, Kempker RR, Tenna A, Smitson C, Berhe N, Fekade D (2013). High prevalence of Cryptococcal antigenemia among HIV-infected patients receiving antiretroviral therapy in Ethiopia. PLoS One.

[44] Osazuwa OF, Dirisu O, Okuonghae E (2012). Cryptococcal antigenemia in anti-retroviral naive AIDS patients: prevalence and its association with CD4 cell count. Acta Med Iran.

[45] Rugemalila J, Maro VP, Kapanda G, Ndaro AJ, Jarvis JN (2013). Cryptococcal antigen prevalence in HIV-infected Tanzanians: a cross-sectional study and evaluation of a point-of-care lateral flow assay. Trop Med Int Health.

[46] Magambo KA, Kalluvya SE, Kapoor SW, Seni J, Chofle AA, Fitzgerald DW (2014). Utility of urine and serum lateral flow assays to determine the prevalence and predictors of cryptococcal antigenemia in HIV-positive outpatients beginning antiretroviral therapy in Mwanza. Tanzania J Int AIDS Soc.

[47] National Institute for Communicable Diseases. Monthly surveillance report - October 2014. 2014.

[48] Pongsai P, Atamasirikul K, Sungkanuparph S (2010). The role of serum cryptococcal antigen screening for the early diagnosis of cryptococcosis in HIV-infected patients with different ranges of CD4 cell counts. J Infect.

[49] Micol R, Lortholary O, Sar B, Laureillard D, Ngeth C, Dousset JP (2007). Prevalence, determinants of positivity, and clinical utility of cryptococcal antigenemia in Cambodian HIV-infected patients. J Acquir Immune Defic Syndr.

[50] Kwan CK, Leelawiwat W, Intalapaporn P, Anekthananon T, Raengsakulrach B, Peters PJ (2014). Utility of cryptococcal antigen screening and evolution of asymptomatic cryptococcal antigenemia among HIV-infected women starting antiretroviral therapy in Thailand. J Int Assoc Provid AIDS Care.

[51] Ganiem AR, Indrati AR, Wisaksana R, Meijerink H, van der Ven A, Alisjahbana B (2014). Asymptomatic cryptococcal antigenemia is associated with mortality among HIV-positive patients in Indonesia. J Int AIDS Soc.

[52] Smith RM, Nguyen TA, Ha HT, Thang PH, Thuy C, Lien TX (2013). Prevalence of cryptococcal antigenemia and cost-effectiveness of a cryptococcal antigen screening program—Vietnam. PLoS One.

[53] Patel S, Shin GY, Wijewardana I, Vitharana SR, Cormack I, Pakianathan M (2013). The prevalence of cryptococcal antigenemia in newly diagnosed HIV patients in a Southwest London cohort. J Infect.

[54] McKenney J, Smith RM, Chiller TM, Detels R, French A, Margolick J (2014). Prevalence and correlates of cryptococcal antigen positivity among AIDS patients—United States, 1986–2012. MMWR Morb Mortal Wkly Rep.

[55] Osazuwa F, Dirisu JO, Okuonghae PE, Ugbebor O (2012). Screening for cryptococcal antigenemia in anti-retroviral naive AIDS patients in Benin City. Nigeria Oman Med J.

[56] Worodria W, Massinga-Loembe M, Mazakpwe D, Luzinda K, Menten J, Van Leth F (2011). Incidence and predictors of mortality and the effect of tuberculosis immune reconstitution inflammatory syndrome in a cohort of TB/HIV patients commencing antiretroviral therapy. J Acquir Immune Defic Syndr.

[57] World Health Organization (2011). Rapid advice: diagnosis, prevention and management of cryptococcal disease in HIV-infected adults, adolescents and children.

[58] Republic of Rwanda Ministry of Health. National guidelines for comprehensive care of people living with HIV in Rwanda. 2011.

[59] Republic of Kenya Ministry of Health. Guidelines for antiretroviral therapy in Kenya. 2011.

[60] Botswana Ministry of Health. Botswana national HIV & AIDS treatment guidelines. 2012.

[61] National Medicine and Therapeutics Policy Advisory Committee (NMTPAC) and the TB and AIDS Directorate Ministry of Health and Child Care. Guidelines for antiretroviral therapy for the prevention and treatment of HIV in Zimbabwe. 2013.

[62] Uganda Ministry of Health. Addendum to the antiretroviral treatment guidelines for Uganda. 2013.

[63] Republic of South Africa Department of Health. National consolidated guidelines for the prevention of mother-to-child transmission of HIV (PMTCT) and the management of HIV in children, adolescents and adults. 2015.

[64] Panel on Opportunistic Infections in HIV-Infected Adults and Adolescents. Guidelines for the prevention and treatment of opportunistic infections in HIV-infected adults and adolescents: recommendations from the Centers for Disease Control and Prevention, the National Institutes of Health, and the HIV Medicine Association of the Infectious Diseases Society of America

[65] Lindsley MD, Mekha N, Baggett HC, Surinthong Y, Autthateinchai R, Sawatwong P (2011). Evaluation of a newly developed lateral flow immunoassay for the diagnosis of cryptococcosis. Clin Infect Dis.

[66.•] Hansen J, Slechta ES, Gates-Hollingsworth MA, Neary B, Barker AP, Bauman S, et al. Large-scale evaluation of the immuno-mycologics lateral flow and enzyme-linked immunoassays for detection of cryptococcal antigen in serum and cerebrospinal fluid. Clin Vaccine Immunol. 2013;20(1):52–5. doi:10.1128/CVI.00536-12. **This article highlights the evaluation of the new lateral flow assay using 589 serum and 411 CSF samples comparing it to the enzyme immunoassay. The lateral flow assay showed excellent concordance with the enzyme immunoassay**. 10.1128/CVI.00536-12 PMC3535775 23114703

[67] Escandon P, Lizarazo J, Agudelo CI, Chiller T, Castaneda E (2013). Evaluation of a rapid lateral flow immunoassay for the detection of cryptococcal antigen for the early diagnosis of cryptococcosis in HIV patients in Colombia. Med Mycol.

[68] Binnicker MJ, Jespersen DJ, Bestrom JE, Rollins LO (2012). Comparison of four assays for the detection of cryptococcal antigen. Clin Vaccine Immunol.

[69] McMullan BJ, Halliday C, Sorrell TC, Judd D, Sleiman S, Marriott D (2012). Clinical utility of the cryptococcal antigen lateral flow assay in a diagnostic mycology laboratory. PLoS One.

[70.•] Jarvis JN, Percival A, Bauman S, Pelfrey J, Meintjes G, Williams GN, et al. Evaluation of a novel point-of-care cryptococcal antigen test on serum, plasma, and urine from patients with HIV-associated cryptococcal meningitis. Clin Infect Dis. 2011;53(10):1019–23. doi:10.1093/cid/cir613. **This article describes the performance of the lateral flow assay using serum, plasma and urine and shows high correlation between the samples. The article also highlights the potential capability of using the test for early detection of cryptococcal disease even in the absence of a lumbar puncture**. 10.1093/cid/cir613 PMC3193830 21940419

[71] Boulware DR, Rolfes MA, Rajasingham R, von Hohenberg M, Qin Z, Taseera K (2014). Multisite validation of cryptococcal antigen lateral flow assay and quantification by laser thermal contrast. Emerg Infect Dis.

[72] Williams D, Kiiza T, Velamakkani S, Kiggundu R, Meya D, Rhein J et al., editors. Evaluating cryptococcal antigen lateral flow assay by fingerstick whole blood in HIV-infected persons with meningitis. ID Week. Philadelphia, PA; 2014.

[73] Kwizera R, Nguna J, Kiragga A, Nakavuma J, Rajasingham R, Boulware DR (2014). Performance of cryptococcal antigen lateral flow assay using saliva in Ugandans with CD4 <100. PLoS One.

[74] Vijayan T, Chiller T, Klausner JD. Sensitivity and specificity of a new cryptococcal antigen lateral flow assay in serum and cerebrospinal fluid. MLO Med Lab Obs. 2013;45(3):16–20. PMC4119400 23822028

[75.•] Rajasingham R, Rolfes MA, Birkenkamp KE, Meya DB, Boulware DR. Cryptococcal meningitis treatment strategies in resource-limited settings: a cost-effectiveness analysis. PLoS Med. 2012;9(9):e1001316. doi:10.1371/journal.pmed.1001316. **This article describes aspects of cost effectiveness using different regimens of cryptococcal meningitis treatment and highlights short-course (7-day) amphotericin induction therapy coupled with high-dose fluconazole (1,200 mg/day) as the most cost-effective regimen**. 10.1371/journal.pmed.1001316 PMC3463510 23055838

[76] Rajasingham R, Boulware DR (2012). Reconsidering cryptococcal antigen screening in the U.S. among persons with CD4 <100 cells/mcL. Clin Infect Dis.

